# FGFR4 Arg388 allele correlates with tumour thickness and FGFR4 protein expression with survival of melanoma patients

**DOI:** 10.1038/sj.bjc.6603181

**Published:** 2006-05-23

**Authors:** S Streit, D S Mestel, M Schmidt, A Ullrich, C Berking

**Affiliations:** 1 Department of Molecular Biology, Max-Planck-Institute of Biochemistry, Am Klopferspitz 18a, Martinsried D-82152, Germany; 2 Department of Dermatology, Ludwig-Maximilian University of Munich, Frauenlobstr. 9-11, Munich D-80337, Germany; 3 Munich Cancer Registry/IBE, Ludwig-Maximilian University of Munich, Marchioninistr. 15, Munich D-81377, Germany

**Keywords:** melanoma, FGFR4 protein expression, FGFR4 Arg388 polymorphism, tumour thickness, survival, microvessel density

## Abstract

A single nucleotide polymorphism in the gene for FGFR4 (−Arg388) has been associated with progression in various types of human cancer. Although fibroblast growth factors (FGFs) belong to the most important growth factors in melanoma, expression of FGF receptor subtype 4 has not been investigated yet. In this study, the protein expression of this receptor was analysed in 137 melanoma tissues of different progression stages by immunohistochemistry. FGFR4 protein was expressed in 45% of the specimens and correlated with pTNM tumour stages (UICC, *P*=0.023 and AJCC, *P*=0.046), presence of microulceration (*P*=0.009), tumour vascularity (*P*=0.001), metastases (*P*=0.025), number of primary tumours (*P*=0.022), overall survival (*P*=0.047) and disease-free survival (*P*=0.024). Furthermore, FGFR4 Arg388 polymorphism was analysed in 185 melanoma patients by polymerase chain reaction-restriction fragment length polymorphism (PCR-RFLP). The Arg388 allele was detected in 45% of the melanoma patients and was significantly associated with tumour thickness (by Clark's level of invasion (*P*=0.004) and by Breslow in mm (*P*=0.02)) and the tumour subtype nodular melanoma (*P*=0.002). However, there was no correlation of the FGFR4 Arg388 allele with overall and disease-free survival. In conclusion, the Arg388 genotype and the protein expression of FGFR4 may be potential markers for progression of melanoma.

Melanoma is one of the most aggressive tumours, once metastasised. The rising incidence makes this cancer an issue of ever increasing clinical and economic importance and research interest (Garland *et al*, 2003; Tucker and Goldstein, 2003; Berwick *et al*, 2005). The identification of new prognostic markers may help to better distinguish low from high-risk disease apart from the classical histopathologic and clinical criteria (Carlson *et al*, 2005) and may offer new targets for therapy.

The mechanism by which normal melanocytes transform to malignant melanoma cells is still poorly understood, but it is known that growth factors and their corresponding receptors play an important role in the progression of melanoma (Shih and Herlyn, 1994; Krasagakis *et al*, 1995). One of the best characterised activators for cell growth, proliferation, differentiation and migration in melanoma is basic fibroblast growth factor (bFGF) (Klagsbrun and Baird, 1991; Bikfalvi *et al*, 1997), a multifunctional cytokine that interacts with four different types of high-affinity receptors (FGFR1-4). These are members of a family of transmembrane receptors with ligand-induced tyrosine kinase activity. Basic fibroblast growth factor is highly mitogenic for melanocytes and is provided by neighbouring keratinocytes and fibroblasts in the skin (Halaban *et al*, 1988a, 1988b; Root and Shipley, 1999). Especially in combination with ultraviolet radiation, bFGF has been shown to act as a potent inductor of melanoma (Berking *et al*, 2001). In contrast to melanocytes, bFGF can be produced by nevus and melanoma cells and may act as an autocrine growth factor through the FGF receptors (Halaban *et al*, 1987; Halaban *et al*, 1988c; Albino *et al*, 1991; Scott *et al*, 1991; Reed *et al*, 1994; al-Alousi *et al*, 1996a, 1996b; Meier *et al*, 2003). It has been demonstrated that melanoma cells cannot survive if bFGF or FGFR1 are targeted (Wang and Becker, 1997; Graeven *et al*, 2001; Valesky *et al*, 2002). Yayon *et al* (1997) demonstrated that direct interference with the activity of FGFR1 suppressed cell proliferation and survival of melanoma possibly through the inactivation of a Src-family kinase. To date bFGF/FGFR1 is the best characterised growth factor/growth factor receptor coexpression pair that has been found in primary and metastatic melanoma (Kaipainen *et al*, 1994; Wang and Becker, 1997). However, bFGF may also act through the other FGF receptor subtypes, which have been described in various human diseases and malignancies.

Genetic alterations, such as point mutations, have been identified in FGFR genes and have been linked to developmental defects as well as neoplastic degeneration (Balasubramanian *et al*, 2002). Mutations in the FGFR1 and FGFR2 genes have been shown to cause craniosynostotic aberrations such as Apert-, Pfeiffer-, Jackson-, Weiss- and Crouzon syndrome (Muenke and Schell, 1995). A single amino-acid exchange of a conserved glycine to arginine in the transmembrane domain of FGFR3 has been found in 90% of patients suffering from achondroplasia (Muenke and Schell, 1995). Other mutations in the FGFR3 or FGFR2 genes have been associated with bladder, cervical or colorectal cancer (Cappellen *et al*, 1999; Jang *et al*, 2001).

Recently, Bange *et al* (2002) discovered a germline polymorphism in the gene encoding for FGFR4. The single nucleotide polymorphisms (SNP) results in a change of the amino-acid sequence at codon 388 from glycine to arginine (Gly388 to Arg388). It was demonstrated that FGFR4 Arg388 was not involved in tumour initiation, as the FGFR4 alleles showed a similar distribution in breast cancer patients and healthy controls and appeared in approximately 50% of the human population. However, the Arg388 isotype was significantly overrepresented in the group of node-positive breast cancer patients with early relapse but not associated with a shortened disease-free survival in node-negative breast cancer (Bange *et al*, 2002). Recently, Streit *et al* (2004) demonstrated an association between high expression of FGFR4 Arg388 allele and poor clinical outcome in head and neck squamous cell carcinoma (HNSCC). These findings were supported by independent groups with similar results in soft tissue sarcoma, prostate cancer and lung adenocarcinoma (Morimoto *et al*, 2003; Wang *et al*, 2004; Spinola *et al*, 2005).

In this study, the focus is on FGFR4 in melanoma analysing its expression and different genotypes in relation with pathological and clinical parameters.

## MATERIALS AND METHODS

### Tissue samples

Formalin-fixed and paraffin-embedded archival tissues of 198 primary melanomas were collected at the Department of Dermatology of the Ludwig-Maximilian University of Munich, Germany. All patients had a histopathologically confirmed diagnosis of melanoma between 1993 and 1999. The median follow-up was 59 months (female 54 months, male 59.5 months; range 2–141 months). The cohort consisted of 93 female and 105 male patients with a median age of 65 years (female 68 years; range 26–92 years, male 62 years; range 23–93 years) at the date of diagnosis. The selected cohort of patients included primary melanomas with low tumour thickness followed by metastasis and/or death of the patients after 5 years follow-up as well as primary melanomas with high tumour thickness, but no metastasis and/or death of the patients after 5 years. In all, 121 patients stayed free of metastases (pTNM stage 1 and 2), 42 patients had local and regional metastases (pTNM stage 3) and 35 patients had distant metastases (pTNM stage 4). Patient characteristics and pTNM tumour stages according to UICC (Union Internationale Contre le Cancer) and AJCC (American Joint Committee on Cancer) are summarised in Table 1.

### Immunohistochemistry

Paraffin-embedded sections (5 *μ*m) were subjected to deparaffinisation in xylene and rehydrated in a graded series of isopropanol. Antigen retrieval was achieved by microwave in citrate buffer, pH 6.0 (Chemicom IHC Select, Temecula, Canada) for FGFR4 and Ki-67 and by Proteinase (P8038, SIGMA, Steinheim, Germany) for CD-31. Blocking of unspecific bindings was done with FCS/Tris 20%. As primary antibodies were used: rabbit anti-human FGFR4 ((C16): sc-124, Santa Cruz Biotechnology, Inc., Heidelberg, Germany), mouse anti-human Ki-67 (M7187, DAKO, Glostrup, Denmark) and mouse anti-human CD-31 (M0823, DAKO, Glostrup, Denmark). They were incubated overnight at room temperature. Secondary antibodies were mouse anti-rabbit (MAR) antibody (M0737, DAKO, Glostrup, Denmark) and alkaline phosphatase-conjugated rabbit anti-mouse (RAM) antibody (Z0259, DAKO, Glostrup, Denmark) followed by alkaline phosphatase anti-alkaline phosphatase (APAAP, Mouse) antibody (D 0651, DAKO). Fast Red was used as substrate and Mayer's hematoxylin as counterstaining. Two investigators (DSM, CB) read all tissue sections using a light microscope (Axilloskop, Leica). Microvessel density (MVD) and microulcerations were recorded in a blinded manner on HE-stained sections. Microvessel density was estimated in the centre and at the edge of each tumour and graded low, medium or high in comparison to the surrounding tissue. Additionally, MVD was evaluated by staining for CD-31 and counting a mean of five visual fields (range 2–10) at × 200 magnification. Likewise, the proliferation rate was evaluated with the help of staining for Ki-67.

### DNA extraction

Five to ten paraffin-embedded sections (10 *μ*m) were deparaffinised with xylene and rehydrated in a graded series of ethanol. The specimens were then treated with proteinase K (Roche, Mannheim, Germany) in digestion buffer (Tris-Cl; EDTA; SDS10%; pH 8.5) for 36–72 h at 55°C. After incubation in phenol and chloroform : isoamylalcohol (24 : 1) and repeated cycles of precipitation, sodium acetate and 100% ethanol were added and incubated at −20°C for 24 h. After centrifugation at 4°C for 30 min, pellets were washed with 70% ethanol and suspended in deionised H_2_O. Samples were stored at 4°C.

### FGFR4 genotyping

The analysis of FGFR4 Arg388 was carried out as described previously (Bange *et al*, 2002). The following primers were used: 5′-GACCGCAGCAGCGCCGAGGCCAG-3′ and 5′-AGAGGGAAGAGGGAGAGCTTCTG-3′. Primers (2 *μ*M) and genomic DNA were combined in a 25-*μ*l total reaction volume using Ready-to-Go PCR beads (Pharmacia, Uppsala, Sweden). After denaturing at 95°C for 3 min, the reaction mixture was subjected to 35 cycles of 45 s at 95°C and 45 s at 72°C followed by one cycle at 72°C for 5 min. The 168 bp fragment was digested overnight with BstNI (NewEngland BioLabs) according to the manufacturer's instruction. Restriction fragments were resolved on a 12% nondenaturing polyacrylamide gel and DNA was visualised with ethidium bromide. The Arg388 allele was characterised by two distinctive fragments of 80 and 29 bp, whereas a single distinctive band of 109 bp was observed for the Gly388 allele.

### Statistical analysis

Statistical analyses were performed using the statistical packages MedCalc (MedCalc Software, Belgium), WinStat (R. Fitch Software, Staufen, Germany) and SPSS (Superior Performance Software System, Munich, Germany). Association between different categorical variables were assessed by Pearson's *χ*^2^ test. Univariate analyses of time to death (overall survival) or time to recurrence (disease-free survival) were performed using the product-limit procedure (Kaplan–Meier method) and compared using log-Rank statistics, with date of histological diagnosis as the starting point. A *P*-value of <0.05 was regarded significant.

## RESULTS

### FGFR4 protein expression in melanoma tissues

**Figure 1:**
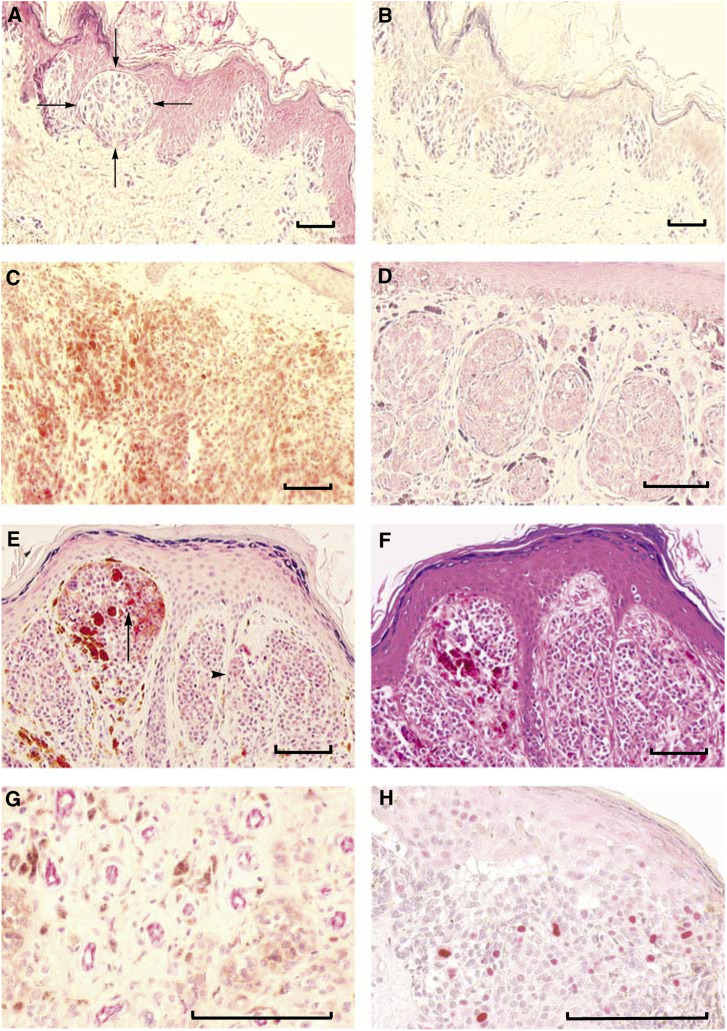
Immunohistochemical analysis of FGFR4, CD-31 and Ki-67 in primary melanoma tissues. (**A**) No expression of FGFR4 in a tumour nest (arrows) of a superficial spreading melanoma (SSM). (**B**) Negative control of **A** after preincubation of the FGFR4 antibody with a blocking peptide. (**C** and **D**) Expression of FGFR4 (red) in a nodular malignant melanoma (NMM) with high intensity (**C**) and in an SSM with intermediate intensity (**D**). (**E**) Expression of FGFR4 in an NMM with areas of high intensity (arrow) and intermediate intensity (arrowhead). (**F**) H&E stained section of **E**. (**G**) Expression of CD-31 in vessels (red) in an FGFR4-positive NMM. (**H**) Expression of Ki-67 (red) in proliferating melanoma cells of an FGFR4-positive SSM. Scale bar, 100 *μ*m.

By immunohistochemical analysis, 137 different primary melanomas could be evaluated for protein expression of FGFR4. In 45% (61/137) of the tumours, FGFR4 was detected with varying staining intensities (Figure 1E) in the cytoplasm of the melanoma cells (Table 2). Strong reactivity for FGFR4 was found in 4 cases (7%; Figure 1C), while 51 tumours (83%) displayed intermediate (Figure 1D) and 6 (10%) only minimal staining intensities. Positive staining was found throughout the tumours and was not different at the infiltrative front or at the margin of ulcerations and necrosis. Besides melanoma cells keratinocytes, fibroblasts, nerves, smooth muscle cells, sweat glands and sebaceous glands were stained for FGFR4, but not melanocytes. The number of FGFR4-expressing melanoma cells per tumour was 100% in 20 cases, 50–99% in 10 cases, 10–49% in 21 cases and 1–9% in 10 cases (Table 2).

### Association of FGFR4 expression with survival of patients

The status of FGFR4 protein expression was correlated with clinical and pathological data (Table 2). There was no obvious correlation between staining intensity and tumour characteristics or patient outcome. Thus, for the following statistical analyses tumours with FGFR4 expression were combined in one group, regardless of the number of positively stained cells and the staining intensity.

No correlation was found between protein expression of FGFR4 and gender, age at date of diagnosis or localisation of the tumour. There was also no association with tumour thickness and histological subtype.

**Figure 2:**
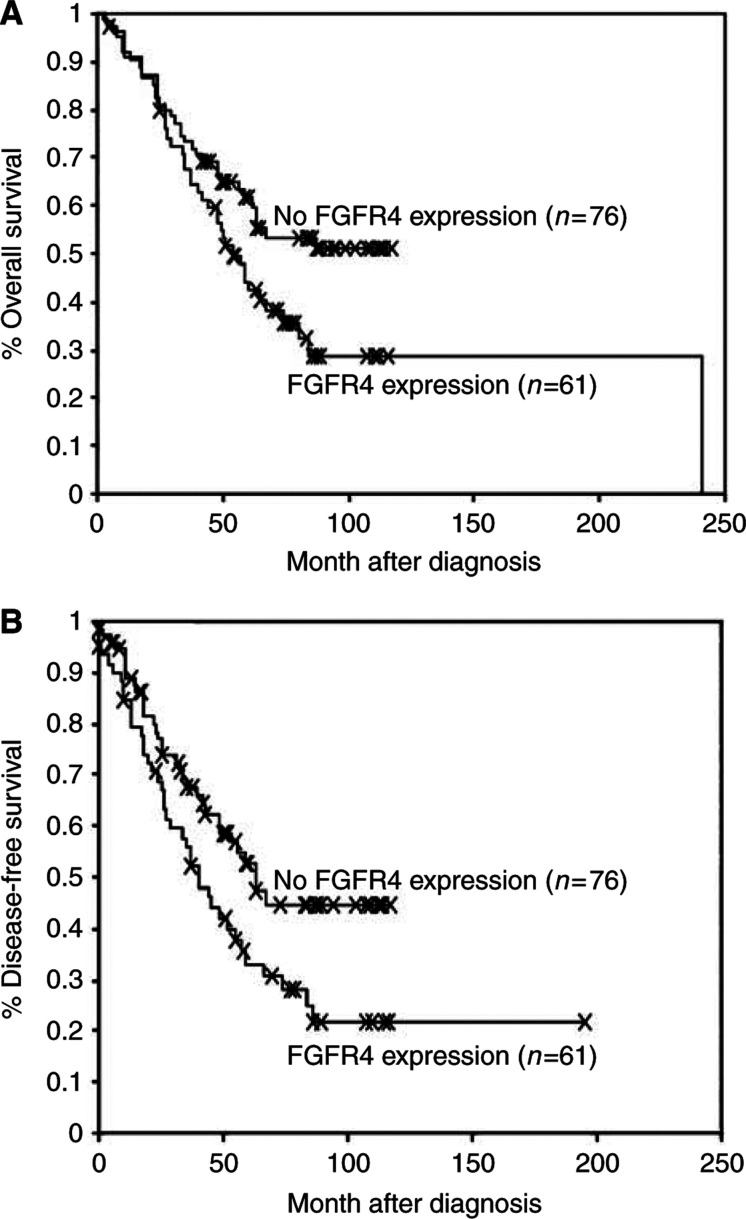
Kaplan–Meier survival curves of patients with positive FGFR4 protein expression *vs* no FGFR4 protein expression in melanomas. End point was (**A**) death or (**B**) relapse/metastases. FGFR4 protein expression is linked with progressive disease.

With respect to the UICC and AJCC TNM staging systems a significant association was found between FGFR4 protein expression and pTNM tumour stages III and IV as opposed to tumour stages I and II (UICC, *P*=0.023 and AJCC *P*=0.046). Furthermore, microscopic ulceration of the tumours, which is a negative prognostic criteria according to the revised AJCC classification, was significantly correlated with positive expression of FGFR4 (*P*=0.009). In addition, 31 of 56 (55%) melanoma patients with metastatic disease showed FGFR4 protein expression (*P*=0.025). When comparing FGFR4 protein expression and number of primary tumours, 22 (65%) of 34 patients with more than one melanoma were positive for FGFR4 (*P*=0.022). Furthermore, Kaplan–Meier survival analysis for a mean follow-up time of 72 months revealed that expression of FGFR4 in melanoma was associated with reduced overall survival (*P*=0.047; Figure 2A) as well as reduced disease-free survival (*P*=0.024; Figure 2B) of the respective patients.

### Association of FGFR4 protein expression with microvessel density and proliferation rate

Tumour vascularity was evaluated on 137 melanoma tissues and compared to the expression of FGFR4. There was a significant correlation between a high MVD and positive expression of FGFR4 (Table 2). This was confirmed by the higher amount of CD-31-positive vessels within the tumours with positive FGFR4 protein expression (Figure 1G) as opposed to FGFR4-negative tumours (Table 2).

Similarly, the proliferation marker Ki-67 was increased in FGFR4-positive tumours (Figure 1H) compared to FGFR4-negative tumours (Table 2). The Ki-67-positive cells were mainly localised to the infiltrative front of the tumours.

### FGFR4 genotype distribution in patients with melanoma and association with clinicopathological parameters

Genotype analysis of the Gly388 allele and the Arg388 allele of FGFR4 was performed in 185 melanomas by polymerase chain reaction-restriction fragment length polymorphism (PCR-RFLP). Gly/Gly, Gly/Arg and Arg/Arg genotypes were detected in 101 (55%), 69 (37%) and 15(8%) cases, respectively.

The Arg388 genotype of FGFR4 was compared with clinical and pathological variables (Table 3). For statistical analysis, patients homozygous or heterozygous for the Arg388 allele were combined into one group.

Regarding the UICC and AJCC TNM staging systems and the Arg388 polymorphism of FGFR4, no significant association could be seen. There was also no correlation of the Arg388 genotype with ulceration, microvessel density and proliferation (Ki-67). Further clinical variables like gender, age at diagnosis and tumour localisation did not show any significant association with FGFR4 Arg388. No correlation of the Arg388 allele was detected with the number of tumours per patient and the presence, number or type of metastases.

However, there was a strong correlation between the presence of at least one FGFR4 Arg388 allele and tumour thickness according to Clark's level of invasion IV and V as opposed to I, II and III (*P*=0.004) and to Breslow's thickness (⩽1 mm *vs* >1 mm; *P*=0.02). When comparing the two most common histological subtypes with each other, namely nodular malignant melanoma (NMM), which represents the more invasive type, and superficial spreading melanoma (SSM), 41 patients (60%) with NMM were carriers of the FGFR4 Arg388 polymorphism (*P*=0.002), whereas only 27 patients (40%) with NMM had the Gly388 genotype.

## DISCUSSION

Under physiological conditions, the activity and the cellular signals of regulated tyrosine kinases (RTKs) are tightly controlled. A dysfunction of these control mechanisms, for example, by an aberrant expression of the RTK/ligand system or genetic alterations, can result in a deregulated tyrosine kinase activity. Such alterations are frequently linked to cancer and other hyperproliferative or developmental disorders. In order to validate the influence of FGFR4 and its Arg388 polymorphism in melanoma, a large patient cohort with long-term follow-up of up to 141 months was evaluated for FGFR4 protein expression in addition to the FGFR4 genotype. The present study demonstrates an association between FGFR4 in melanoma and clinical and histopathologic parameters indicative of progression.

FGFR4 was expressed in 45% of 137 primary melanomas with different intensities. High expression of FGFR4 has been described already for breast cancer (Lehtola *et al*, 1992; Jaakkola *et al*, 1993), pancreatic cancer (Leung *et al*, 1994) and renal cell carcinoma (Takahashi *et al*, 1999). The cytoplasmic staining of the FGFR4 in melanoma cells is in agreement with other publications investigating the expression for FGFR4 in other types of cancer (Gowardhan *et al*, 2005; Mawrin *et al*, 2005). In contrast to FGFR1, no nuclear staining could be observed for FGFR4 (Maher, 1996; Stachowiak *et al*, 1997).

Comparing the expression with clinical data of melanoma patients there was a significant correlation between FGFR4 protein expression and pTNM stage according to UICC and AJCC classification. The majority of early-stage melanoma did not express FGFR4 in contrast to advanced-stage melanomas. Consistent with these results an association between ulceration of melanoma and FGFR4 protein expression was found, linking FGFR4 protein expression with worse prognosis as has been shown for ulcerations (Balch *et al*, 2000). These results suggest that FGFR4 protein expression may be a prognostic marker for worse clinical outcome. This is supported by our additional findings that FGFR4 was significantly overrepresented in the group of melanoma patients with metastases. Moreover, a significant association between FGFR4 protein expression and microvessel density was observed and an increased number of proliferating Ki-67-positive melanoma cells was found at the edges and at the infiltrating front of FGFR4-postive tumours. The presence of FGFR4 may facilitate the proliferative and angiogenic effects of bFGF and thus confer an increased metastatic potential to the melanoma cells (Rak and Kerbel, 1997; Wang and Becker, 1997; Compagni *et al*, 2000; Straume and Akslen, 2002). It has been demonstrated before that increased tumour vascularity plays a decisive role in the prognosis of melanoma patients (Kashani-Sabet *et al*, 2002).

Our data lead to the conclusion that FGFR4 protein expression is linked with tumour progression. This hypothesis is further supported by the analysis of patient's outcome in relation to FGFR4 expression by Kaplan–Meier method revealing a correlation between FGFR4 and poor survival rates. Based upon our results of this experimental study, it would be interesting to conduct a new investigation on a representative cohort of melanoma patients in order to determine, in a multivariate analysis, the impact of FGFR4 on prognosis of melanoma patients in comparison to other known prognostic factors.

Furthermore, patients with FGFR4-expressing tumours had more often additional melanomas in the past. This may hint to a role of FGFR4 in melanoma development, even though the mechanism is unknown.

Regarding the genotypes of FGFR4 in melanoma patients, 55% (101/185) had a homozygous Gly allele, while 45% (84/185) showed at least one Arg388 allele. A similar distribution of the Gly388 and Arg388 alleles was reported for breast cancer patients (Bange *et al*, 2002).

Notwithstanding the molecular mechanisms by which the FGFR4 Arg388 polymorphism leads to a more aggressive clinical phenotype is not completely understood. However, it has been shown that cancer cells ectopically expressing FGFR4 Arg388 possessed increased cell motility as well as invasiveness (Bange *et al*, 2002; Wang *et al*, 2004). This is in agreement with our observations that the Arg388 allele was predominantly found in nodular melanoma, which is characterised by vertical growth and increased risk for metastasis compared to SSM. In accordance with this, the Arg388 genotype was also associated with tumour thickness by Clark's level and by Breslow. On the other hand, we could not find any correlation between the Arg388 genotype and decreased survival rates of patients. Thus, the FGFR4 Arg388 genotype may be used as an additional risk factor for tumour progression with respect to invasion; however, it does not seem to be a good prognostic factor for disease outcome. It remains to be speculated if a larger cohort of patients or a longer follow-up period would change these results.

In accordance with previous studies, no correlation was found between the genotype and the protein expression of FGFR4 (Streit *et al*, 2004).

In conclusion, the previously observed genotype-dependent difference in disease progression suggests that FGFR4 Arg388 as a marker for tumour progression in different cancers can only partly be demonstrated in melanoma. Nevertheless, independently from this specific polymorphism, FGFR4 seems to play an important role in melanoma considering the high expression rates in advanced tumours and the positive correlation with worse clinical outcome.

## Figures and Tables

**Table 1:** Patient characteristics

**Patients**		***n*=198**
Sex		
Male		105 (53%)
Female		93 (47%)
		
Median age at diagnosis (years)		65 (range 23–93)
Date of diagnosis		1993–1999
		
*pTNM UICC staging (n=198)*	0: Tis, N0, M0	0 (0%)
pT1: ⩽0.75 mm	Ia: pT1, N0, M0	57 (29%)
pT2: 0.76–1.5 mm	Ib: pT2, N0, M0	20 (10%)
pT3a: 1.51–3.0 mm or level IV	IIa: pT3, N0, M0	34 (17%)
pT3b: 3.01–4.0 mm or level IV	IIb: pT4a, N0, M0	10 (5%)
pT4a: >4.0 mm or level V	IIIa: pT4a/pT4b, N1, M0	29 (15%)
pT4b: Satellite met(s) within 2 cm from tumour	IIIb: any pT, N1/N2, M0	13 (6%)
N1: Lymph node met(s) ⩽3 cm	IV: any pT, any N, M1	35 (18%)
N2a: Lymph node met(s) >3 cm		
N2b: In-transit met(s)		
N2c: Lymph node met(s) >3 cm and in-transit met(s)		
M: Distant met(s)		
		
*pTNM AJJC staging (n=139)*	0: Tis, N0, M0	0 (0%)
T1: ⩽1.0 mm	Ia: T1a, N0, M0	39 (28%)
a: without ulceration; b: with ulceration or level IV or V	Ib: T1b, T2a, N0, M0	18 (13%)
T2: 1.01–2.0 mm	IIa: T2b, T3a, N0, M0	12 (8%)
a: without ulceration; b: with ulceration	IIb: T3b, T4a, N0, M0	13 (9%)
T3: 2.01–4.0 mm	IIc: T4b, N0, M0	5 (4%)
a: without ulceration; b: with ulceration	IIIa: T1-4a, N1a/N2a, M0	11 (7%)
T4: >4.0 mm	IIIb: T1-4b, N1a/N2a, N1b/N2b	18 (13%)
a: without ulceration; b: with ulceration	T1-4a/b, N2c, M0	
N1: One lymph node	IIIc: T1-4b, N1b/N2b/N3, M0	5 (4%)
a: micrometastasis; b: macrometastasis	IV: any T, any N, M1	22 (16%)
N2: 2–3 lymph nodes		
a: micrometastasis; b: macrometastasis		
c: in-transit met(s)/satellite(s) without metastatic lymph nodes		
N3: ⩾4 lymph nodes, satellite(s) and metastatic lymph node(s)		
M1: Distant met(s)		
		
Median follow-up time (months)		59 (range 2–141)

Abbreviations: AJCC, American Joint Committee on Cancer; met(s), metastases; pTNM, tumour, lymph node and distant metastasis status; UICC, Union Internationale Contre le Cancer.

**Table 2:** FGFR4 protein expression and clinical/pathological variables

**FGFR4 expression**	**Yes (%)**	**No (%)**	***P*-value**
Patients *n*=137	*n*=61 (45)	*n*=76 (55)	
Median age (years)	70	62	
			
*Age at diagnosis (years)*			
⩽55	14 (23)	25 (33)	NS
>55	47 (77)	51 (67)	
			
*Sex*			
Male	33 (24)	42 (31)	NS
Female	28 (20)	34 (25)	
			
*Localisation of tumour*			
Extremities	29 (21)	55 (40)	NS
Trunk, head/neck	27 (20)	26 (19)	
			
*Staining intensity*			
Minimal	6 (10)		NS
Intermediate	51 (83)		
Strong	4 (7)		
			
*Positively stained cells per tumour*			
1–9%	10 (16)		NS
10–49%	21 (34)		
50–99%	10 (16)		
100%	20 (34)		
			
*Microvessel density (n=137)*			
Low	24 (18)	57 (42)	0.001
Medium	21 (15)	12 (9)	
High	15 (11)	8 (6)	
			
CD-31 (mean^a^; *n*=17)	44	28	
Ki-67 (mean^b^; *n*=17)	24	14.5	
			
*pTNM UICC staging (n=137)*			
Ia	15 (11)	23 (17)	NS
Ib	4 (3)	11 (8)	(0.023 when stages III+
IIa	8 (6)	12 (9)	
IIb	3 (2)	5 (4)	IV compared to I+II)
IIIa	11 (8)	12 (9)	
IIIb	5 (4)	5 (4)	
IV	15 (11)	8 (6)	
			
*pTNM AJCC staging (n=137)*			
Ia	13 (9)	24 (18)	NS
Ib	6 (4)	10 (7)	(0.046 when stages III+IV compared to I+II)
IIa	3 (2)	8 (6)	
IIb	5 (4)	8 (6)	
IIc	3 (2)	2 (1)	
IIIa	5 (4)	6 (4)	
IIIb	8 (6)	9 (7)	
IIIc	3 (2)	2 (1)	
IV	14 (10)	8 (6)	
			
*Tumour thickness Breslow*			
⩽1 mm	27 (20)	32 (23)	NS
>1 mm	34 (25)	44 (32)	
			
*Clark level*			
I	0	0	NS
II	14 (10)	14 (10)	
III	18 (13)	28 (20)	
IV	26 (19)	30 (22)	
V	3 (2)	4 (3)	
			
*Ulceration (n=137)*			
Yes	27 (20)	18 (13)	0.009
No	33 (24)	59 (43)	
			
*No. of primary tumours*			
1	39 (28)	64 (47)	0.022
>1	22 (16)	12 (9)	
			
*Metastasis*			
Yes	31 (23)	25 (18)	0.025
No	30 (22)	51 (37)	
			
*No. of metastases*			
1	13 (23)	12 (22)	NS
>1	18 (32)	13 (23)	
			
*Subtype of metastasis*			
Local	11 (36)	12 (48)	NS
Regional	5 (16)	5 (20)	
Distant	15 (48)	8 (32)	
			
*Tumour subtype*			
SSM	30 (22)	37 (27)	NS
NMM	25 (18)	25 (18)	
Others	6 (5)	14 (10)	

Abbreviations: AJCC, American Joint Committee on Cancer; NMM, nodular malignant melanoma; No., number; NS, not significant; pTNM, tumour, lymph node and distant metastasis status; SSM, superficial spreading melanoma; UICC, Union Internationale Contre le Cancer.

a Vessels per visual field.

b Cells per visual field.

**Table 3:** FGFR4 Arg388 genotype and clinical/pathological variables

**FGFR4 allele**	**G/G (%)**	**G/R (%)**	**R/R (%)**	***P*-value**
Patients *n*=185	*n*=101	*n*=69	*n*=15	
Median age (years)	68	64	55	
				
*Age at diagnosis*				
⩽55	28 (28)	19 (28)	8 (53)	NS
>55	73 (72)	50 (72)	7 (47)	
				
*Sex*				
Male	53 (29)	33 (18)	12 (6)	NS
Female	48 (26)	36 (19)	3 (2)	
				
*Localisation of tumour*				
Extremities	64 (35)	49 (26)	5 (3)	NS
Trunk, head/neck	37 (20)	20 (11)	10 (5)	
				
*Microvessel density (n=139)*				
Low	45 (32)	33 (24)	7 (5)	NS
Medium	15 (11)	12 (9)	4 (3)	
High	12 (9)	10 (7)	1 (1)	
				
CD-31 (mean^a^; *n*=17)	38	30	26	
Ki-67 (mean^b^; *n*=17)	18	14	41	
				
*pTNM UICC staging (n=185)*				
Ia	32 (16)	19 (10)	3 (2)	NS
Ib	11 (6)	5 (3)	2 (1)	
IIa	13 (7)	17 (9)	3 (2)	
IIb	6 (3)	4 (2)	0 (0)	
IIIa	14 (7)	11 (6)	2 (1)	
IIIb	4 (2)	6 (3)	1 (1)	
IV	21 (11)	7 (4)	4 (2)	
				
*pTNM AJCC staging (n=139)*				
Ia	21 (15)	15 (11)	3 (2)	NS
Ib	13 (9)	4 (3)	1 (1)	
IIa	4 (3)	7 (5)	1 (1)	
IIb	6 (4)	5 (4)	2 (1)	
IIc	2 (1)	3 (2)	0 (0)	
IIIa	4 (3)	5 (4)	2 (1)	
IIIb	10 (7)	7 (5)	1 (1)	
IIIc	0 (0)	3 (2)	0 (0)	
IV	14 (10)	4 (3)	2 (1)	
				
*Tumour thickness Breslow*				
⩽1 mm	50 (27)	26 (14)	4 (2)	0.02
>1 mm	51 (28)	43 (23)	11 (6)	
				
*Clark level*				
I	0 (0)	0 (0)	0 (0)	NS
II	24 (12)	17 (9)	2 (1)	(0.004 when
III	39 (20)	10 (5)	6 (3)	levels IV+V
IV	37 (19)	36 (18)	5 (3)	compared to
V	1 (1)	6 (3)	2 (1)	II+III)
				
*Ulceration (n=139)*				
Yes	23 (17)	19 (14)	3 (2)	NS
No	51 (37)	34 (24)	9 (6)	
				
*No. of primary tumours*				
1	75 (41)	55 (30)	14 (7)	NS
>1	26 (14)	14 (7)	1 (1)	
				
*Metastasis*				
Yes	39 (21)	24 (13)	7 (4)	NS
No	62 (34)	45 (24)	8 (4)	
				
*No. of metastases*				
1	19 (27)	7 (10)	3 (4)	NS
>1	20 (29)	17 (24)	4 (6)	
				
*Type of metastasis*				
Local	14 (36)	11 (46)	2 (29)	NS
Regional	4 (10)	6 (25)	1 (14)	
Distant	21 (54)	7 (29)	4 (57)	
				
*Tumour type*				
SSM	56 (30)	26 (14)	5 (3)	0.002
NMM	27 (15)	32 (17)	9 (5)	
Others	18 (10)	11 (6)	1 (1)	

AJCC, American Joint Committee on Cancer; NMM, nodular malignant melanoma; No., number; NS, not significant; pTNM, tumour, lymph node and distant metastasis status; SSM, superficial spreading melanoma; UICC, Union Internationale Contre le Cancer; .

a Vessels per visual field.

b Cells per visual field.
